# Biochemical and Structural Characterization of the Interaction between the Siderocalin NGAL/LCN2 (Neutrophil Gelatinase-associated Lipocalin/Lipocalin 2) and the N-terminal Domain of Its Endocytic Receptor SLC22A17*

**DOI:** 10.1074/jbc.M115.685644

**Published:** 2015-12-03

**Authors:** Ana-Isabel Cabedo Martinez, Katharina Weinhäupl, Wing-Kee Lee, Natascha A. Wolff, Barbara Storch, Szymon Żerko, Robert Konrat, Wiktor Koźmiński, Kathrin Breuker, Frank Thévenod, Nicolas Coudevylle

**Affiliations:** From the ‡ Department of Computational and Structural Biology, Max F. Perutz Laboratories, University of Vienna, Campus Vienna Biocenter 5, 1030 Vienna, Austria,; § Chair of Physiology, Pathophysiology, and Toxicology and ZBAF, Faculty of Health, School of Medicine, Witten/Herdecke University, Stockumer Strasse 12, 58453 Witten, Germany,; ¶ Institute of Organic Chemistry and Center for Molecular Biosciences Innsbruck, University of Innsbruck, CCB, Innrain 80/82, 6020 Innsbruck, Austria, and; ‖ Faculty of Chemistry, Biological and Chemical Research Centre, University of Warsaw, Żwirki i Wigury 101, 02-089 Warsaw, Poland

**Keywords:** cell surface protein, intrinsically disordered protein, membrane protein, protein complex, protein-protein interaction, intrinsically disordered protein, lipocalin 2, neutrophil gelatinase-associated lipocalin receptor, fuzzy complex

## Abstract

The neutrophil gelatinase-associated lipocalin (NGAL, also known as LCN2) and its cellular receptor (LCN2-R, SLC22A17) are involved in many physiological and pathological processes such as cell differentiation, apoptosis, and inflammation. These pleiotropic functions mainly rely on NGAL's siderophore-mediated iron transport properties. However, the molecular determinants underlying the interaction between NGAL and its cellular receptor remain largely unknown. Here, using solution-state biomolecular NMR in conjunction with other biophysical methods, we show that the N-terminal domain of LCN2-R is a soluble extracellular domain that is intrinsically disordered and interacts with NGAL preferentially in its apo state to form a fuzzy complex. The relatively weak affinity (≈10 μm) between human LCN2-R-NTD and apoNGAL suggests that the N terminus on its own cannot account for the internalization of NGAL by LCN2-R. However, human LCN2-R-NTD could be involved in the fine-tuning of the interaction between NGAL and its cellular receptor or in a biochemical mechanism allowing the receptor to discriminate between apo- and holo-NGAL.

## Introduction

The neutrophil gelatinase-associated lipocalin (NGAL,^3^ also known as siderocalin, lipocalin 2 (LCN2), or 24p3) is a mammalian lipocalin that has the remarkable ability to bind the bacterial siderophore enterobactin (Ent) with a very high affinity (0.5 nm) (1). This unique feature endows NGAL with bacteriostatic properties that are mainly active during the antibacterial innate immune response. During a bacterial infection, NGAL will sequester enterobactin, withdrawing the siderophore from the bacterium thereby limiting its access to environmental iron. Along this line it has been shown that mouse LCN2 (mLCN2) is overexpressed during the early stage of a bacterial infection (2) and that mLCN2-deficient mice are significantly more sensitive to bacterial infections (3). The physiological implications of the bacteriostatic properties of NGAL as well as the biochemistry of its interaction with siderophores have been intensively investigated and are very well characterized (4–6).

In addition to its bacteriostatic properties, NGAL has been shown to be involved in many other physiological and pathological processes, suggesting that NGAL carries pleiotropic functions. Indeed, NGAL seems to play a role in organogenesis and cell differentiation (7, 8), cell migration, apoptosis, and inflammation (9, 10). Moreover, increasing evidence suggests that NGAL is also involved in cancer progression (11) and metastasis (12).

However, the biochemical mechanisms supporting these pleiotropic functions remain unclear. NGAL's ability to protect the matrix metalloprotease-9 (MMP9) from degradation by forming a MMP9·NGAL complex (13) could be the biochemical basis underlying NGAL's involvement in organogenesis and cancer progression but cannot satisfactorily account for NGAL's contribution to cell differentiation, apoptosis, and inflammation. Therefore, it has been proposed that the siderophore-mediated iron transport properties of NGAL could play a role in these processes. This potential mode of action has been supported by the identification of a cellular receptor for NGAL (NGAL-R/hLCN2-R in humans or 24p3-R/mLCN2-R/rLCN2-R in mouse and rat, also called SLC22A17), which promotes the endocytosis of both free and iron-bound NGAL, together with the discovery of small molecules that may act as mammalian siderophores and can preserve the iron binding properties of NGAL in the absence of bacterial infection (14, 15). It has been shown that using these mammalian siderophores, NGAL can transport iron in and out of the cell via its endo- and exocytosis by mLCN2-R (7, 8) and thus regulates the expression of iron responsive genes.

Despite the important body of literature establishing the involvement of NGAL and LCN2-R in inflammation and cancer, little is known about the biochemistry of the interaction between NGAL and its cellular receptor. It has been shown that 24p3-R binds NGAL with a very high affinity (*K_D_* ≈ 90 pm) (16) and that 24p3-R also exhibits high avidities for various ligands (such as transferrin, albumin, or metallothionein) (17–20). However, in a recent paper, Correnti *et al.* (21) questioned whether NGAL is truly able to shuttle iron in and out of the cell based on 1) their observation that gentisic acid (the putative mammalian siderophore) could not form a stable ternary complex with NGAL and iron and 2) their inability to demonstrate any physical interaction between NGAL and mLCN2-R.

**FIGURE 1.**
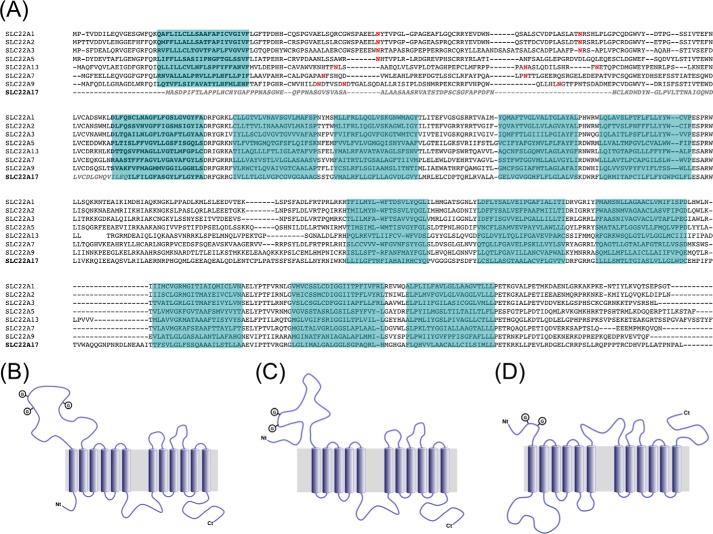
*A*, sequence alignments of SLC22 family members. Trans-membrane segments are highlighted in *blue* based on the TMHMM server predictions; *N*-glycosylation sites are colored in *red*. The sequence of the hLCN2-R-NTD construct used in this study is in *italics* and colored in *light gray. B*, canonic topology of the SLC22 family of organic cation transporters. *C*, proposed topology of LCN2-R. *D*, alternative topology proposed by Devireddy *et al.* (15).

In an attempt to clarify these contradicting reports we undertook a nuclear magnetic resonance (NMR)-based biochemical study of the interaction between NGAL and its putative cellular receptor LCN2-R. LCN2-R belongs to the SLC22 family of organic ion transporters (22). This type of transmembrane protein is typically involved in the transport of small charged or polar molecules and usually consists of 12 transmembrane (TM) helical segments organized in two bundles of six TMs each connected by a large intracellular loop (Fig. 1*A*) (23). SLC22 transporters also contain a large soluble extracellular domain located between TM1 and TM2 that generally includes two-three *N*-glycosylation sites and an intracellular C-terminal domain (Fig. 1*B*). Sequence alignments reveal that LCN2-R, however, exhibits atypical features, as the first TM is not present, and the sequence of the 100 following residues, until TM2, significantly differs from the canonical SLC22 sequence (Fig. 1*A*). This results in an unusual putative topology for SLC22A17 where the first 100 residues would form an extracellular soluble domain containing two *N*-glycosylation sites followed by a first bundle of five TMs, a large intracellular loop, and final bundle of six TMs, and an intracellular C-terminal domain (Fig. 1*C*).

Because the full-length receptor is hardly amenable to solution state NMR, we decided to focus on the 105-residue N-terminal domain (hLCN2-R-NTD) and the role it could possibly play in the interaction between NGAL and its cellular receptor. Therefore, we present here the cloning, expression, purification, and the biochemical characterization of the soluble and, presumably, extracellular N-terminal domain of hLCN-2R as well as its interaction mode with NGAL.

## Experimental Procedures

### 

#### 

##### Expression and Purification of hLCN2-R-NTD

The coding region for the first 105 resides of hLCN-2R (hLCN2-R-NTD) was amplified from commercial cDNA by PCR and inserted in the bacterial expression vector pET-M11, yielding pET-M11-hLCN2-R-NTD, to encode hLCN2-R-NTD fused to an N-terminal His_6_ tag plus the TEV cleavage site. The quadruple mutant (hLCN2-R-NTD-QM) was generated by sequentially replacing the cysteines 15, 59, 70, and 94 by serines using the QuikChange mutagenesis kit from Stratagene. The same expression and purification protocol was followed for both the wild-type and quadruple mutant hLCN2-R-NTD.

The protein was expressed in the *Escherichia coli* strain T7 Express (New England BioLabs). hLCN2-R-NTD expression was induced at an *A*_600 nm_ of 0.8 by the addition of 0.8 mm isopropyl 1-thio-β-d-galactopyranoside. The cells were collected after 16 h of expression at 30 °C by centrifugation at 6000 rpm for 10 min and resuspended in 30 ml of ice-cold lysis buffer (20 mm NaP_i_, pH 7.4, 50 mm NaCl) per liter of the original bacterial culture. Bacteria were lysed by passing through a French press, and the cell lysate was cleared by centrifugation at 18,000 rpm for 20 min. The supernatant was discarded, and the pellet containing the protein was resuspended in 7 m urea. The resulting solution was loaded onto a Ni^2+^-loaded HiTrap 5-ml affinity column (GE Healthcare), washed with 2 column volumes of high salt buffer (7 m urea, 1.5 m NaCl, 10 mm imidazole), and eluted with high imidazole buffer (7 m urea, 50 mm NaCl, 0.5 m imidazole). The protein-containing fractions were collected, and hLCN2-R-NTD was refolded via buffer exchange by dialysis against the TEV cleavage buffer (20 mm NaP_i_, pH 7.4, 50 mm NaCl, 0.5 mm EDTA) in the presence of 2 mm DTT. After three successive dialysis steps, 1 mg of TEV protease was added for each 50 mg of hLCN2-R-NTD. The cleavage of the His_6_ tag was carried at 4 °C overnight. The sample was subsequently loaded onto a Superdex 75 HiLoad 16/60 preparation grade gel filtration column (GE Healthcare) equilibrated with 20 mm Tris, pH 7.4, 50 mm NaCl, 1 mm DTT. The protein-containing fractions were collected and concentrated.

##### Expression of NGAL and Preparation of Its Paramagnetic Form

The coding region for human NGAL (hNGAL) was amplified by PCR from pGEX-4T-hNGAL(C87S) vector (1) and inserted in the bacterial expression vector pET-M11, yielding pET-M11-hNGAL(C87S), encoding hNGAL(C87S) fused to an N-terminal His_6_ tag plus the TEV cleavage site. To link the spin label for paramagnetic relaxation enhancement studies, the naturally occurring cysteine in position 87 was reintroduced using the QuikChange mutagenesis kit from Stratagene leading to the wild-type form of hNGAL containing a unique cysteine. The hNGAL(C87S)_GFP fusion protein used for thermophoresis experiments was obtained by inserting in-frame the coding sequence for a short linker and the GFP after the coding sequence of hNGAL into pET-M11-hNGAL(C87S). All hNGAL forms were expressed and purified as follows.

Proteins were expressed in the *E. coli* strain BL21(DE3) pLysS. hNGAL expression was induced at an *A*_600 nm_ of 0.8 by the addition of 0.8 mm isopropyl 1-thio-β-d-galactopyranoside. The cells were collected after 16 h of expression at 30 °C by centrifugation at 6000 rpm for 10 min and resuspended in 30 ml of ice-cold lysis buffer (20 mm NaP_i_, pH 7.4, 50 mm NaCl) per liter of the original bacterial culture. Bacteria were lysed by passing through a French press, and the cell lysate was cleared by centrifugation at 18,000 rpm for 20 min. The supernatant containing the soluble protein fraction was loaded onto a Ni^2+^-loaded HiTrap 5 ml affinity column (GE Healthcare), washed with 2 column volumes of high salt buffer (20 mm NaP_i_, pH 7.4, 1.5 m NaCl, 10 mm imidazole), and eluted with high imidazole buffer (20 mm NaP_i_, pH 7.4, 50 mm NaCl, 0.5 m imidazole). The hNGAL-containing fractions were collected, and the buffer was exchanged by dialysis to the TEV cleavage buffer (20 mm NaP_i_, pH 7.4, 50 mm NaCl, 0.5 mm EDTA, 1 mm DTT). 1 mg of TEV protease was added for each 50 mg of hNGAL. The cleavage of the His_6_ tag was carried out at 4 °C overnight. The sample was subsequently loaded onto a Superdex 75 HiLoad 16/60 prep grade gel filtration column (GE Healthcare) equilibrated with 20 mm Tris pH 7.4, 50 mm NaCl, 1 mm DTT. To avoid any co-purified ligand, all samples were unfolded by 6 m guanidinium hydrochloride and subsequently loaded on a desalting column to separate the polypeptide chain from any ligand. The protein samples were then refolded by several dialysis steps against 20 mm Tris, pH 7.4, 50 mm NaCl, 1 mm DTT. The correct refolding of the protein was monitored by ^1^H,^15^N HSQC.

Enterobactin and the NGAL/[Fe^III^(Ent)]^3−^ were prepared as described elsewhere (24). In the case of the MTSL-tagged samples, the MTSL was attached to cysteine 87 following a protocol described elsewhere (25).

##### Mass Spectrometry (MS)

Disulfide bond analysis of hLCN2-R-NTD utilized a 7 T Fourier transform ion cyclotron resonance instrument equipped with an electrospray ionization (ESI) source (Bruker, Vienna, Austria). Protein was desalted as described previously (26) and electrosprayed from 1 μm solutions in 1:1 H_2_O/CH_3_OH at pH 2.5 (adjusted by the addition of CH_3_COOH). The reduction reaction was performed with 20 μm protein and 2.5 mm 1,4-dithiothreitol (DTT) in H_2_O at 60 °C and quenched after variable reaction times *t* by lowering the solution pH to 2.5 by the addition of CH_3_COOH and cooling to room temperature.

##### Disulfide Bond Reduction Kinetics

MS of hLCN2-R-NTD utilized a 7 T Fourier transform ion cyclotron resonance instrument equipped with an ESI source (Bruker). Proteins were desalted as described previously (26) and electrosprayed from 1 μm solutions in 1:1 H_2_O/CH_3_OH at pH 2.5 (adjusted by the addition of CH_3_COOH). From ESI MS spectra with internal calibration using polyethylene glycol with an average molecular mass of ∼1000 (PEG 1000), the measured mass (most abundant isotopic peak) of hLCN2-R-NTD after refolding and removal of the N-terminal His_6_ tag and the TEV cleavage site by TEV protease was 11,050.20 Da, which agrees to within 0.8 ppm with the calculated mass of the 106-residue protein with two disulfide bonds (most abundant isotopic peak, 11050.21 Da). A quantitative analysis of the measured, and calculated isotopic profiles revealed that two disulfide bonds were formed in >99% of the protein (2S ensemble), and one disulfide bond was formed in the remaining fraction (<1%, 1S ensemble).

##### Isothermal Titration Calorimetry (ITC)

Binding of free and bound NGAL to hLCN2-R-NTD was determined by ITC using a Microcal ITC200 microcalorimeter. Experiments were carried out at 25 °C in 20 mm Tris, pH 7.4, 50 mm NaCl. The reference cell contained Milli-Q water. The concentration of hLCN2-R-NTD in the reaction cell was 50 μm. The concentration of NGAL in the syringe was 500 μm. The titration consisted of 19 successive injections of 4 μl, with a stirring speed of 800 rpm, separated by intervals of 300 s. Data analysis was done with the Origin software assuming a single binding site.

##### NMR Measurements

All NMR samples were concentrated up to 0.5 mm protein in 20 mm Tris, 50 mm NaCl, pH 7.4, supplemented with 10% D_2_O. NMR experiments were carried out at 25 °C on Varian Inova spectrometers operating at 500, 600, or 800 MHz. All spectra were processed using NMRPipe/NMRDraw (27) and analyzed with Sparky and CARA (28).

##### NMR Resonance Assignment

All spectra were acquired at 298 K on an Agilent Direct Drive 2 600 MHz spectrometer using the standard 5-mm ^13^C,^1^H,^15^N triple-resonance probe head. The backbone ^1^H, ^13^C, and ^15^N resonances were assigned using sparse random sampling of indirectly detected time domains in order to increase resolution. A three-dimensional HNCO experiment was used as a base spectrum for SMFT (sparse multidimensional Fourier transform) processing of higher dimensionality experiments (29). Sampling artifacts from three-dimensional HNCO were removed using cleaner3d program (iterative algorithm of discrete Fourier transform for processing randomly sampled NMR data sets (30)). Backbone assignment was achieved using five-dimensional HN(CA)CONH (29) and (HACA)CON(CA)CONH (31). Side-chain assignments were obtained using five-dimensional HabCabCONH (29) and HC(CC-tocsy)CONH experiments (32).

All NMR data sets were processed by multidimensional Fourier transformation using the home-written software package (Cent3) (32, 33). The resonance assignment was performed using the TSAR program (34). The input data for TSAR was prepared using Sparky software.

##### Relaxation Dispersion Measurements

Backbone ^15^N single-quantum relaxation dispersion experiments were carried out at 25 °C on Varian Inova spectrometers operating at 600 and 800 MHz. CPMG-based radio frequency field strengths (ν_CPMG_) ranged from 40 to 960 Hz with a CPMG delay of 40 ms. Duplicate data sets were recorded at selected ν_CPMG_ values for error analysis. All spectra were processed using NMRPipe/NMRDraw (27) and analyzed with Sparky. Relaxation data were analyzed using rdnmr (M. Tollinger, University of Innsbruck) following the already described procedure (35, 36).

##### Antibodies

The rabbit polyclonal antibodies α-NT-24p3-R and α-CT-24p3-R against rat LCN2-R (rLCN2-R were custom made by ImmunoGlobe GmbH (Himmelstadt, Germany) and have been described elsewhere (see Table 1) (19). The rabbit polyclonal antibody PAB20130 directed against a recombinant peptide in the center of the hLCN2-R protein (RWLIVKRQIEEAQSVLRILAERNRPHGQMLGEEAQEALQDLENTCPLPATSSFSFASLLN) and the rabbit polyclonal antibody PAB13044 directed against a synthetic peptide corresponding to the C-terminal 14 amino acids of human SLC22A17 were purchased from Abnova Corp. (Taiwan), and were both shown to react with rLCN2-R (see “Results”). Anti-FLAG M2 mouse monoclonal antibody was from Sigma. The following fluorescently labeled secondary antibodies were used: Alexa Fluor 488-conjugated chicken anti-rabbit IgG and goat anti-mouse IgG (Molecular Probes) and Cy3-conjugated donkey anti-mouse and donkey anti-rabbit IgG (Jackson ImmunoResearch Europe Ltd., Newmarket, UK).

##### rLCN2-R Expression Plasmid

Subcloning of full-length rLCN2-R (GenBank^TM^ accession number NM_177421.3) into the expression vector pcDNA3.1+ (Invitrogen) has been previously described (19). In-frame addition of the FLAG epitope (DYKDDDDK) at the C terminus was carried out by site-directed mutagenesis (QuikChange II site-directed mutagenesis kit, Agilent Technologies Inc.) as per the manufacturer's instructions using the following 5′-GCCACTCCTAATCCTGCCCTCGACTACAAGGACGACGATGACAAGTAA GCAGCCTCTGAGCCTGGTGG-3′ (forward) and 5′-CCACCAGGCTCAGAGGCTGCTTACTTGTCATCGTCGTCCTTGTAGTCGAGGGCAGGATTAGGAGTGGC-3′ (reverse) and 18 cycles of 30 s at 95 °C, 60 s at 55 °C, and 8 min at 68 °C.

##### Cell Culture and Transfection

Immortalized murine distal convoluted tubule cells (mDCT209) and CHO-K1 cells were cultured as previously described (19). Murine cortical collecting duct (mCCDcl1) cells were a kind gift from Dr. E. Hummler (Dept. of Pharmacology and Toxicology, Faculty of Biology and Medicine University of Lausanne, Switzerland) and were cultured according to Fila *et al.* (37). Transient transfection of CHO-K1 cells was carried out with Lipofectamine2000 (Life Technologies) 24 h after plating using a 1:2.5 plasmid DNA:Lipofectamine2000 ratio according to the manufacturer's instructions. The medium was changed 6 h post transfection, and the cells were incubated for an additional 42 h before immunofluorescence staining.

##### Immunofluorescence Microscopy

For fixed cell staining, cells were immediately fixed with 4% paraformaldehyde, then permeabilized with 1% SDS, blocked with 1% BSA, immunostained, and subsequently imaged as described previously (38). For live cell surface staining, cells were first blocked with 1% BSA, immunostained at 4 °C, then paraformaldehyde fixed followed by Hoechst-33342 nuclear counterstaining where applicable (data not shown), as described elsewhere (19). For live staining of mCCDcl1 cells only, antibody incubations were carried out for 30 min at room temperature. α-NT- and α-CT-24p3-R antibodies were used at 5 μg/ml, PAB20130 at 1 μg/ml, PAB13044 at 10 μg/ml, and anti-FLAG M2 at 20 μg/ml. Alexa Fluor 488-conjugated secondary antibodies were diluted 1:500, Cy3-conjugated antibodies 1:600.

##### Microscale Thermophoresis

Microscale thermophoresis measurements (39, 40) were performed on a NanoTemper Monolith NT.115 instrument (NanoTemper Technologies GmbH, Munich, Germany), as previously described (19, 20). For the experiment purified proteins were diluted in 20 mm Tris/HCl, pH 7.4, combined at a fixed concentration of GFP-hLCN2-R-NTD (500 nm) and varying concentrations of NGAL or NGAL/[Fe^III^(Ent)]^3−^ (0–300 μm), allowed to reach steady state, and loaded into standard treated capillaries (catalog #K002) by capillary action. Receptor/ligand interactions were measured at 20% light-emitting diode (LED) for fluorescence detection, and 40% IR laser was used to create a temperature gradient and induce movement of the molecules/complexes driven by thermophoretic force. Measurements were performed at 25 °C with an IR laser on for 30 s and off for 5 s.

## Results

### 

#### 

##### Topological Study of LCN2-R

**FIGURE 2.**
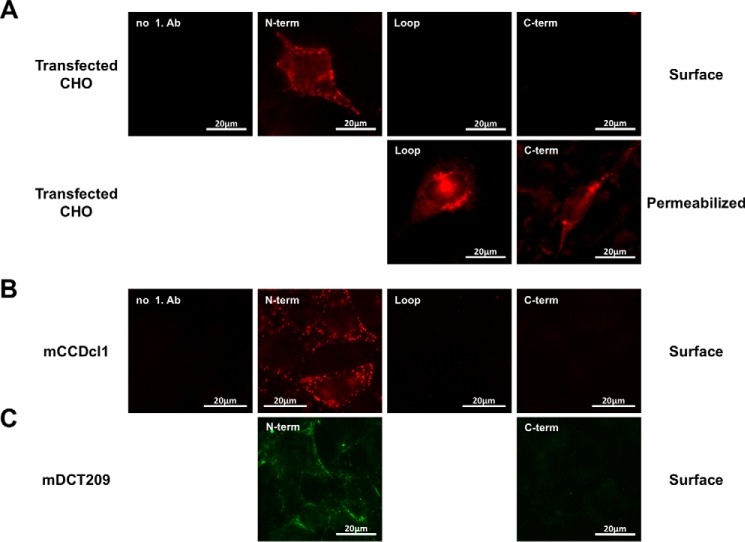
*A*, surface staining of CHO-K1 cells overexpressing FLAG-tagged rLCN2-R. Live or fixed and permeabilized cells were stained with antibodies against the N terminus (α-NT-24p3R), the C terminus (PAB13044), and the loop region between the two N- and C-terminal halves of the protein (PAB20130). Fixed and permeabilized cells were also stained with an antibody against the C-terminal FLAG tag (M2) (data not shown). *B* and *C*, surface staining of cultured cortical collecting duct (mCCDcl1) (*B*) and murine distal convoluted (mDCT209) cells (*C*) expressing mLCN2-R on their surface with α-NT-24p3R (*B* and *C*), PAB13044 (*B*), or α-CT-24p3R (*C*).

Sequence alignment of SLC22A17 with other transporters of the SLC22A family strongly suggests that LCN2-R adopts an unusual topology where the first 100 residues (LCN2-R-NTD) would form a soluble extracellular domain. However, in the topology originally proposed by Devireddy *et al.* (7) the soluble extracellular N-terminal portion extends up to residue 33 followed by TM1 (predicted as a soluble segment in the other topology) and a relatively large intracellular loop (Fig. 1*D*). Noteworthy, this alternative topology is also characterized by an extracellular large loop between the two transmembrane domain bundles as well as an extracellular C-terminal domain. To clarify which of these topologies is the one adopted by LCN2-R *in vivo*, we first performed immunofluorescence staining of CHO-K1 cells transiently transfected with rLCN2-R and immunostained for LCN2-R using antibodies against the N or C terminus as well as against the peptide sequence linking the two transmembrane domain bundles (Table 1). Fig. 2*A* demonstrates that only the N terminus is extracellular by staining of cells that were not fixed and permeabilized (surface staining), which strongly supports the topology proposed in Fig. 1*C*. This was confirmed by surface staining of live cultured murine distal convoluted tubule (mDCT209) and cortical collecting duct (mCCDcl1) cells expressing mLCN2-R using polyclonal antibodies against the N- and C-terminal domains of LCN2-R. Fig. 2, *B* and *C*, reiteratively demonstrate that only the N terminus is extracellular.

**TABLE 1:** **Alignment of immunizing peptides for SLC22A17 antibodies used with human, mouse, and rat SLC22A17** Amino acids that vary between species are in bold.

Antibody	Accession number	α-NT-24p3-R	α-CT-24p3-R PAB13044 (Abnova)	PAB20130 (Abnova)
Immunizing peptide		GA**L**PPNASGWEQPPNS	CDHVPLLATPNPAL	RWLIVKRQIEEAQSVLRILAERNRPHGQMLGEEAQEAL**QD**LENTCPLP**A**TS**S**FSFASLLN
hSLC22A17 isoform b	NM_016609.4	GA**F**PPNASGWEQPPN**A**	CDHVPLLATPNPAL	RWLIVKRQIEEAQSVLRILAERNRPHGQMLGEEAQEAL**QD**LENTCPLP**A**TS**S**FSFASLLN
hSLC22A17 isoform a	NM_020372.3	GA**F**PPNASGWEQPPN**A**	CDHVPLLATPNPAL	RWLIVKRQIEEAQSVLRILAERNRPHGQMLGEEAQEAL**QD**LENTCPLP**A**TS**S**FSFASLLN
mSLC22A17 CRA_a	CH466535.2	GA**L**PPNASGWEQPPNS	CDHVPLLATPNPAL	RWLIVKRQIEEAQSVLRILAERNRPHGQMLGEEAQEAL**HE**LENTCPLP**A**TS**T**FSFASLLN
rSLC22A17	NM_177421.3	GA**L**PPNASGWEQPPNS	CDHVPLLATPNPAL	RWLIVKRQIEEAQSVLRILAERNRPHGQMLGEEAQEAL**QE**LENTCPLP**T**TS**T**FSFASLLN

##### Expression and Purification of the N-terminal Domain of hLCN2-R

**FIGURE 3.**
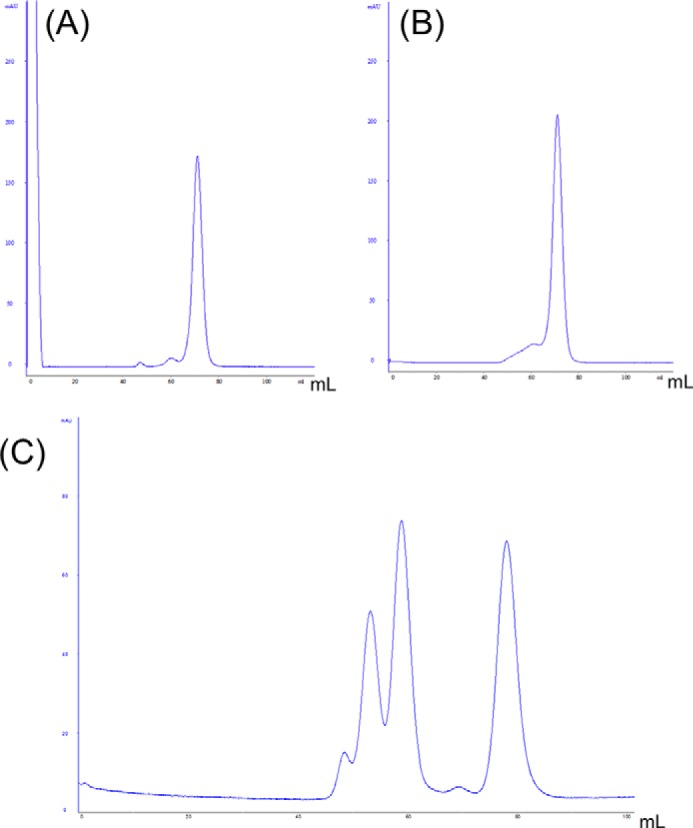
Size exclusion chromatogram (280 nm absorption) of wild-type hLCN2-R-NTD (*A*) and hLCN2-R-QM (*B*). Samples where loaded on top of a Superdex 75 16/60 column and eluted by a flow of 1 ml·min^−1^ of 20 mm Tris, pH 7.4, 20 mm NaCl. *C*, calibration chromatogram, by order of elution: 67 kDa at 53 ml, 43 kDa at 59 ml, 13.7 kDa at 78 ml.

To explore the possibility that the N-terminal domain of LCN2-R is involved in NGAL binding/recognition and based on the topology described in Fig. 1*C*, we cloned and expressed the first 105 residues of hLCN2-R (referred to as hLCN2-R-NTD). After expression, the protein was found in inclusion bodies that were collected and could be resuspended in 7 m urea. After purification in denaturing conditions, hLCN2-R-NTD could subsequently be refolded through an extensive dialysis in the presence of a reducing agent (2 mm DTT). The necessity for a reducing agent in the refolding buffer strongly suggests that the four cysteines present in hLCN2-R-NTD (in positions 17, 61, 72, and 96) have to form the appropriate disulfide bridges to obtain a properly folded and soluble protein. After refolding, the protein is pure, soluble, and appears on the size exclusion chromatography profile (Fig. 3*A*) to have an hydrodynamic radius consistent with a monomeric intrinsically disordered protein (IDP) of this molecular weight (41).

##### Determination of the Disulfide Bond Organization

**FIGURE 4.**
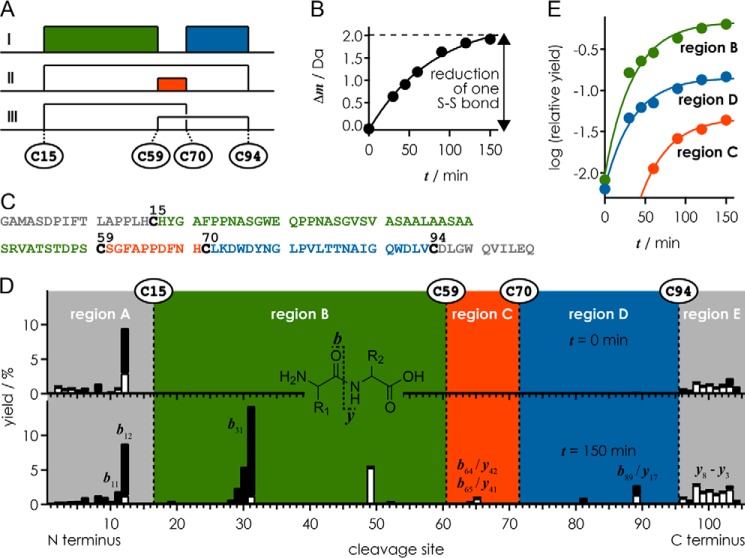
*A*, possible disulfide bond patterns between Cys-15, Cys-59, Cys-70, and Cys-94. *B*, increase in average protein mass *versus* reduction time *t. C*, amino acid sequence (106 residues) of hLCN2-R-NTD after removal of the N-terminal His_6_ tag and the TEV cleavage site. *D*, yield of ***b*** (*black bars*) and ***y*** (*white bars*) fragments from backbone amide bond cleavage (see *inset*) *versus* cleavage site at reduction times 0 and 150 min. *E*, relative yield of the most abundant fragments from cleavage in regions *B*, *C*, and *D versus* reduction time.

From ESI MS spectra with internal calibration using polyethylene glycol with an average molecular weight of ∼1000, the measured mass (most abundant isotopic peak) of hLCN2-R-NTD after refolding and removal of the N-terminal His_6_ tag and the TEV cleavage site by TEV protease was 11,050.20 Da, which agrees to within 0.8 ppm with the calculated mass of the 106-residue protein with two disulfide bonds (most abundant isotopic peak, 11,050.21 Da). A quantitative analysis of the measured isotopic profile revealed that two disulfide bonds were formed in >99% of the protein (2S ensemble), and one disulfide bond was formed in the remaining fraction (<1%, 1S ensemble). To elucidate the connectivity between Cys-15, Cys-59, Cys-70, and Cys-94 (Fig. 4*A*), the kinetics of disulfide bond reduction by DTT was monitored by ESI MS and collision-activated dissociation. After 150 min of reduction, the average protein mass was increased by 1.92 Da compared with that at *t* = 0 min, corresponding to 95% reduction of one disulfide bond (Fig. 4*B*); data at *t* > 150 min could not be recorded because of protein degradation at prolonged reaction times.

The mass values of the ***b***- and ***y***-type fragments from backbone amide bond cleavage by collision-activated dissociation confirmed the amino acid sequence (Fig. 4*C*). Under the low energy conditions used for collision-activated dissociation of the (M+7H)^7+^ ions at *m*/*z* ∼1580 (17 V, corresponding to 119 eV laboratory frame energy), >99% of the ***b*** and ***y*** fragments came from cleavage in regions A and E (sites 1–15 and 94–105) that cannot be bridged by disulfide bonds (Fig. 4*D*), consistent with the disulfide bonds preventing separation of fragments from cleavage in regions B (sites 15–58), C (sites 60–69), and D (sites 71–93) (42). This together with the fact that the ***b*** and ***y*** fragments from cleavage at sites 15–94 (<1%, 1S ensemble) were exclusively from regions B and D, but not from region C, immediately rules out disulfide bond pattern I (Fig. 4*A*).

At *t* = 150 min, additional ***b*** and ***y*** fragments from cleavage in regions B, C, and D were observed (Fig. 4*D*). Yields of the most abundant fragments in regions B (***b***_31_), C (***b***_64_, ***b***_65_, ***y***_41_, ***y***_42_), and D (***b***_89_, ***y***_17_) relative to those in regions A and E (***b***_11_, ***b***_12_, ***y***_3_–***y***_8_) were used to analyze disulfide bond connectivity. For reduction times up to *t* = 45 min, no ***b*** and ***y*** fragments from cleavage in region C were observed, whereas the relative yield of fragments from cleavage in regions B and D steadily increased over the entire time range studied (Fig. 4*E*). This rules out pattern II (Fig. 4*A*), as reduction of both the Cys-15/Cys-70 and Cys-59/Cys-94 bonds, which would be required for the appearance of fragments from cleavage in both regions B and D, would at the same time liberate fragments from cleavage in region C. Instead, the simultaneous appearance of ***b*** and ***y*** fragments from cleavage in regions B and D together with the delayed appearance of fragments from cleavage in region C at *t* = 60 min reveals the disulfide bonding pattern of Cys-15 with Cys-94 and Cys-59 with Cys-70 (*pattern II*, Fig. 4*A*) and shows that the Cys-15–Cys-94 bond is reduced before the Cys-59–Cys-70 bond.

##### Characterization of the Interaction with NGAL by ITC and Microscale Thermophoresis (MST)

**FIGURE 5.**
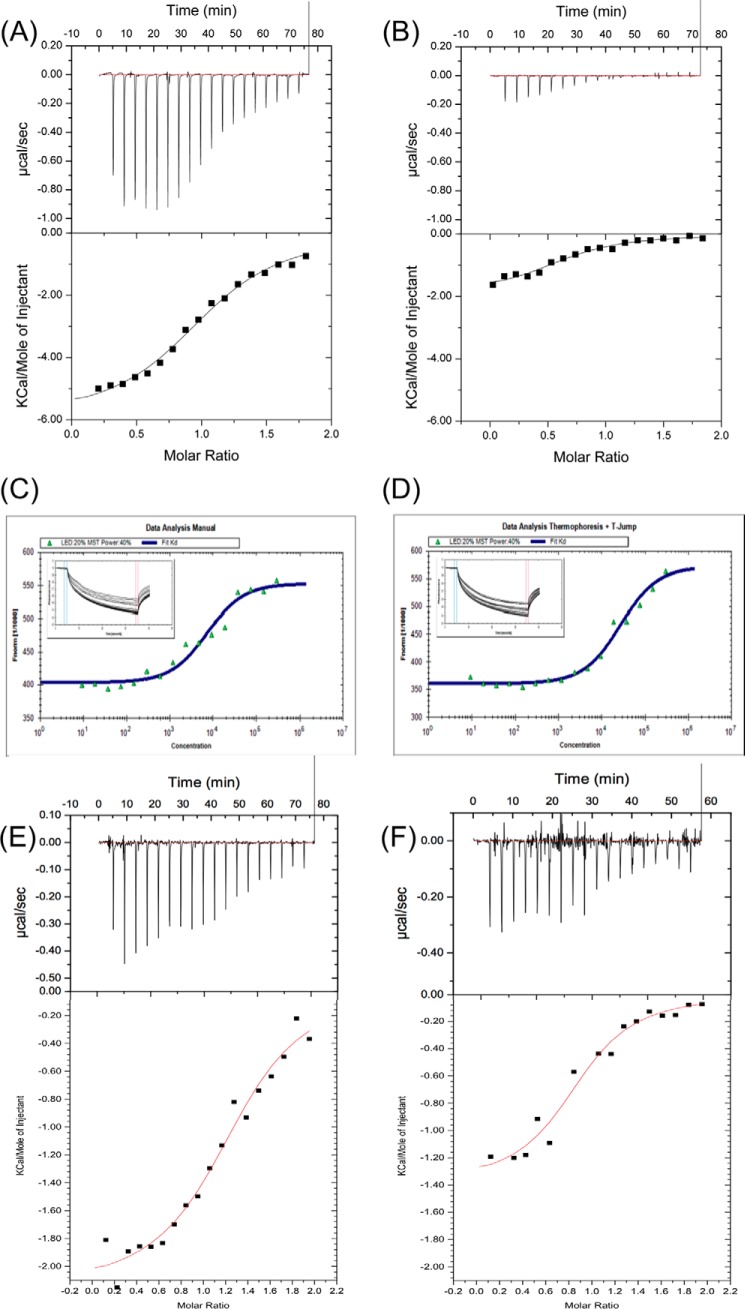
Isothermal titration calorimetry traces of hLCN2-R-NTD binding to apoNGAL (*A*) and (*B*) NGAL/[Fe^III^(Ent)]^3−^. Microscale thermophoresis traces of GFP-hLCN2-NTD binding to apoNGAL (*C*) and NGAL/[Fe^III^(Ent)]^3−^ (*D*). ApoNGAL binds to GFP-hLCN2-NTD with a *K_D_* of 6.6 ± 1.3 μm and NGAL/[Fe^III^(Ent)]^3−^ with a *K_D_* of 20.7 ± 3.5 μm (mean ± S.E. of 8 experiments). Isothermal titration calorimetry traces of hLCN2-R-NTD-DM (*E*) or hLCN2-R-NTD-QM (*F*) binding to apoNGAL.

Next, to determine whether hLCN2-R-NTD can interact with NGAL, we first used ITC. As can be seen in Fig. 5*A*, hLCN2-R-NTD binds to NGAL with an affinity (*K_D_*) of 10 μm and a stoichiometry of 1 to 1 (Table 2). The thermodynamic parameters of binding (Δ*H* = −4.5 kcal·mol^−1^; −*T*Δ*S* = −2.3 kcal·mol^−1^ at 25 °C) reveal that the binding is enthalpically driven but still includes a non-negligible entropic contribution. Noteworthy, after being loaded with ferric-enterobactin ([Fe^III^(Ent)]^3−^), NGAL is no longer able to interact with hLCN2-R-NTD (Fig. 5*B*), suggesting that enterobactin and hLCN2-R-NTD bind to the same site on NGAL (presumably the calix). In parallel to ITC, we also measured the affinity between NGAL and hLCN2-R-NTD using MST. The thermophoresis traces (Fig. 5, *C* and *D*) show that hLCN2-R-NTD binds to apoNGAL with an affinity of ∼7 μm but that hLCN2-R-NTD binding to NGAL/[Fe^III^(Ent)]^3−^ is ∼3× weaker, affinities that are consistent with the ITC results (Table 2).

**TABLE 2:** **Affinities of hLCN2-R for different forms of NGAL measured by ITC and MST** Means ± S.E. of eight experiments are shown for MST and two experiments for ITC. ND, not determined.

Form of NGAL	ITC	MST
	μ*m*	μ*m*
NGAL	10 ± 2	6.6 ± 1.3
NGAL/[Fe^III^(Ent)]^3−^	ND	20.7 ± 3.5

##### NMR Analysis of hLCN2-R-NTD

**FIGURE 6.**
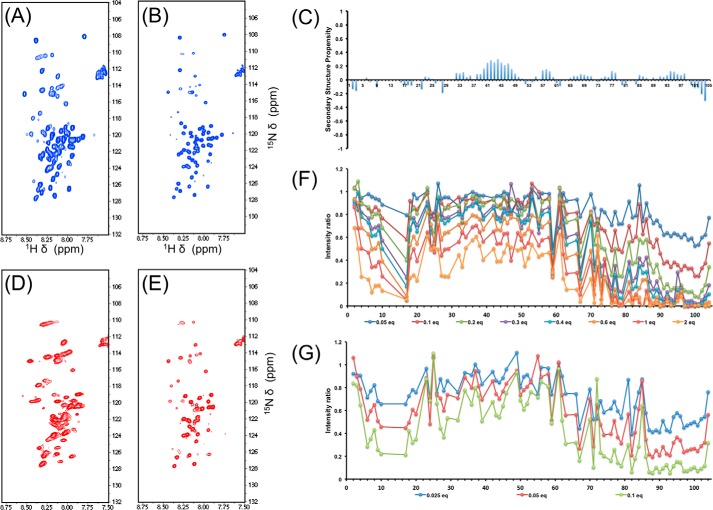
*A*, ^1^H,^15^N HSQC of hLCN2-R-NTD. *B*, ^1^H,^15^N HSQC of hLCN2-R-NTD-QM. *C*, secondary structure propensity of NGAL-R-NTD-QM. *D*, ^1^H,^15^N HSQC of hLCN2-R-NTD in the presence of saturating concentration of NGAL. *E*, ^1^H,^15^N HSQC of hLCN2-R-NTD-QM in the presence of saturating concentration of NGAL. *F*, intensity ratio of hLCN2-R-NTD ^1^H,^15^N HSQC resonances in the presence of increasing NGAL concentrations. *G*, intensity ration of hLCN2-R-NTD ^1^H,^15^N HSQC resonances in the presence of increasing MTSL-tagged NGAL concentrations.

Next we used multidimensional heteronuclear solution state NMR to gain further dynamic and structural insight into the binding mode between NGAL and hLCN2-R-NTD. We expressed hLCN2-R-NTD in minimal media to obtain uniformly ^15^N-labeled hLCN2-R-NTD samples. The ^1^H,^15^N HSQC spectrum of hLCN2-R-NTD (Fig. 6*A*) exhibits the distinctive narrow ^1^H^N^ dispersion of IDPs. Surprisingly, the spectrum also exhibits unusually broad resonances for an IDP, which would indicate that hLCN2-R-NTD, although devoid of any stable secondary or tertiary structures, does not benefit from the fast local dynamics commonly found in IDPs but experiences significant conformational exchange on the micro-to-millisecond timescale. As shown above, hLCN2-R-NTD contains two disulfide bridges: Cys-15–Cys-94, which bridges the N- and C-terminal extremities of the chain, and Cys-59–Cys-70 (Fig. 4*D*). These two disulfide bridges could be at the origin of the broad resonances observed on the ^1^H,^15^N HSQC spectrum either by imposing structural and dynamic restrains or due to micro to millisecond rearrangement of the disulfide bridges pattern, although our mass spectrometry analysis suggests that the disulfide bridges pattern is fairly homogeneous.

Consequently, we designed a quadruple mutant of hLCN2-R where the four cysteine residues were replaced by serines (hLCN2-R-NTD-QM). Size exclusion chromatography shows that hLCN2-R-NTD-QM exhibits the same hydrodynamic radius as the wild-type protein (Fig. 3*B*). We also verified by ITC that the loss of the disulfide bridges does not affect the binding to NGAL (Fig. 5, *E* and *F*). Interestingly, the affinity remains unchanged between the wild type and the different mutants (*K_D_* =9 μm for hLCN2-R-NTD-DM where Cys-15–Cys-94 is mutated out and *K_D_* = 8 μm for hLCN2-R-NTD-QM, where all cysteines are mutated to serines). However, when the number of disulfide bridges decreases, the entropic contribution increases (−*T*Δ*S* = −4.7 kcal·mol^−1^ for hLCN2-R-NTD-DM and −*T*Δ*S* = −5.5 kcal·mole^−1^ for hLCN2-R-NTD-QM) at the expense of the enthalpic contribution (Δ*H* = −2.1 kcal·mol^−1^ for hLCN2-R-NTD-DM and Δ*H* = −1.4 kcal·mole^−1^ for hLCN2-R-NTD-QM). The ^1^H,^15^N HSQC spectrum of the hLCN2-R-NTD-QM mutant is almost identical to the one of the wild type (Fig. 6*B*) with the remarkable difference that many broad peaks in the wild-type spectrum appear narrow in the mutant spectrum. Nevertheless, even for the quadruple mutant, many peaks remain relatively broad indicating that hLCN2-R contains regions of relatively high heterogeneity even in the absence of the conformational heterogeneity generated by the disulfide bridges.

We produced ^13^C,^15^N uniformly labeled hLCN2-R-NTD to perform a sequence-specific resonance assignment. Unfortunately, the poor quality of the three-dimensional spectra did not allow us to achieve a satisfactory level of assignment. To circumvent this problem, we used the hLCN2-R-NTD-QM in conjunction with sparse random sampling of indirectly detected time domains to increase resolution. Backbone assignment was achieved using five-dimensional spectra. Following this strategy, we were able to assign 83% of backbone ^15^N, 92% of ^1^HN, 83% of ^13^Cα, 85% of ^1^Hα, 80% of ^13^Cβ, and 83% of ^13^C′. Additionally, HC(CC-tocsy)CONH spectra allowed the assignment of several side-chain atoms. The neighbor-corrected structural propensity index (43) clearly shows that hLCN2-R-NTD-QM is devoid of any significant secondary structure element (Fig. 6*C*). Finally, because of the high similarity between the two spectra, we were able to transpose the assignment of hLCN2-R-NTD-QM to the spectrum of hLCN2-R-NTD.

##### Interaction between NGAL and hLCN2-R-NTD Monitored by NMR

**FIGURE 7.**
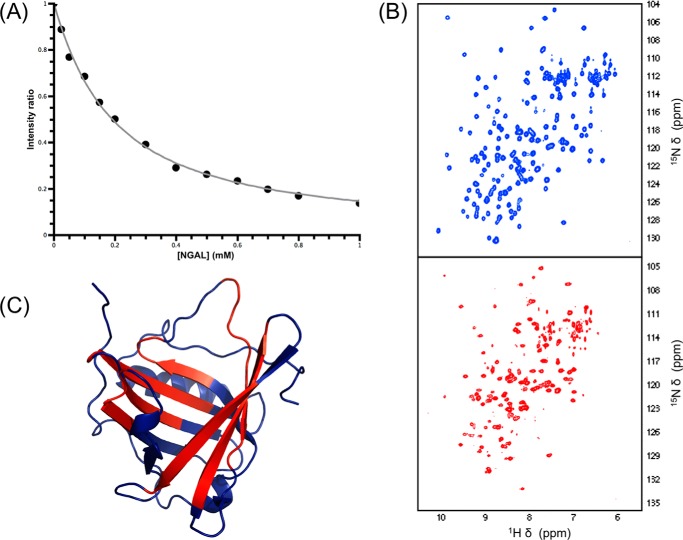
*A*, change in intensity ratio for Ala-10 of hLCN2-R-NTD upon the addition of increasing concentrations of unlabeled NGAL. *B*, ^1^H,^15^N HSQC of NGAL in its free (*blue resonances*) and hLCN2-R-NTD bound form (*red resonances*). *C*, resonance intensity changes upon hLCN2-R-NTD binding mapped on the NGAL three-dimensional structure; residues experiencing a significant chemical shift change upon binding are highlighted in *red*.

Once the assignment of hLCN2-R-NTD is known, we can use NMR to probe the interaction between NGAL and hLCN2-R-NTD with atomic resolution. When performing a ^1^H,^15^N HSQC-based titration of ^15^N-labeled hLCN2-R-NTD with unlabeled NGAL many resonances disappear upon the addition of unlabeled NGAL for both the wild type (Fig. 6*D*) and the quadruple mutant (Fig. 6*E*), whereas only very limited chemical shift changes can be observed. This type of signal decrease is often observed in complexes involving one or more disordered partners, hampering the direct characterization of the bound state. However, the signal disappearance upon the addition of the partners still allows identification of the binding site. Indeed, the effect is strongest for the C-terminal part of hLCN2-R-NTD where most of the resonances completely vanished upon the addition of only 0.3 eq of NGAL (Fig. 6*F*). For the rest of the protein, most resonances remain visible even in presence of 2 eq. of unlabeled NGAL, although the N-terminal part (which is in proximity to the C terminus via the Cys-15–Cys-94 disulfide bridge) seems more affected than the middle of the protein. By following the intensity decrease upon the addition of NGAL for the residues of the N-terminal part (which are strongly affected but are still visible at the end of the titration), we could estimate that the affinity of binding is in the low micromolar range (Fig. 7*A*), which is in agreement with our ITC and MST measurements (see Fig. 5).

The same observation is made with the reciprocal experiment. When unlabeled hLCN2-R-NTD is added to labeled NGAL, the siderocalin experiences only limited chemical shift changes but suffers from severe line broadening (Fig. 7*B*). Mapping the most affected (disappearing) residues upon the addition hLCN2-R-NTD on the three-dimensional structure of NGAL (Fig. 7*C*) confirms that hLCN2-R-NTD seems to bind in the calyx of NGAL (as suggested by the fact that NGAL/[Fe^III^(Ent)]^3−^ is not or only poorly able to interact with hLCN2-R-NTD).

##### Characterization of the Disordered NGAL·hLCN2-R-NTD Complex

To gain more insight into the nature of the disorder leading to the extreme line broadening upon formation of the NGAL·hLCN2-R-NTD complex, we first used paramagnetic relaxation enhancement (44). It has been shown that intermolecular paramagnetic relaxation enhancement can be successfully used for the detection and visualization of lowly populated intermediates in biomolecular complexes. To generate a paramagnetic species, we reintroduced in NGAL the naturally occurring cysteine residue in position 86, to which we attached a spin label (MTSL). The position is outside of the NGAL binding cavity and, therefore, should not affect the binding to hLCN2-R-NTD, but it is close enough to the binding site to generate intermolecular paramagnetic relaxation enhancements. When adding unlabeled MTSL-tagged NGAL to ^15^N labeled hLCN2-R-NTD, it can clearly be seen that the resonances of the C-terminal part of hLCN2-R-NTD are the most affected (Fig. 6*G*), with a decrease in intensity in the presence of 0.1 eq of MTSL-tagged NGAL similar to the intensity decrease observed in presence of 0.2–0.3 eq of diamagnetic NGAL. This clearly indicates that hLCN2-R-NTD experiences paramagnetic relaxation enhancement due to the spatial proximity with the paramagnetic NGAL occurring in the complex formation. The N-terminal part of hLCN2-R-NTD is also significantly affected, which confirms its spatial proximity with the C-terminal part and, therefore, with NGAL while in the complex. More surprising is the enhanced intensity decrease observed for the middle part of the protein, which would indicate that this part interacts at least transiently with the paramagnetic NGAL.

**FIGURE 8.**
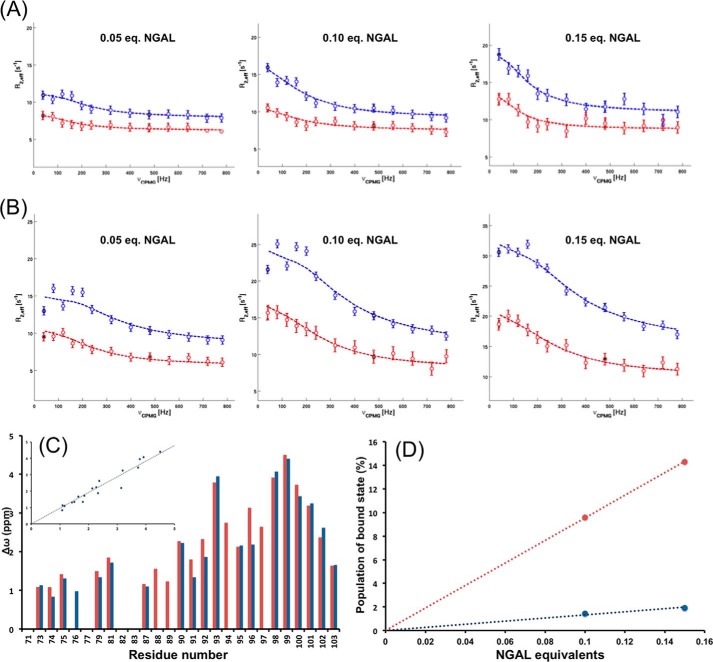
Relaxation dispersion curves measured at 500 (*red circles*) and 800 MHz (*blue circles*) of two residues situated in the binding region of hLCN2R-NTD, theoretical fits for two-site exchange model are shown as *dashed lines. A*, residue 81 with 0. 05, 0.1, and 0.15 eq NGAL. *B*, residue 100 with 0.05, 0.1, and 0.15 eq NGAL. *C*, relaxation dispersion derived Δω for the C-terminal part of hLCN2-R-NTD in the presence of 0.1 (*red bars*) and 0.15 (*blue bars*) eq of NGAL. The *inset* shows the correlation between the two conditions, the correlation coefficient is 0.93. *D*, *blue data points*, relaxation dispersion derived population of bound state as a function of equivalents of NGAL; *red data points*, Expected population of bound state (based on the experimentally measured affinity) as a function of equivalents of NGAL.

Next we exploited the fast exchange between the free and bound form of hLCN2-R-NTD in the presence of substoichiometric amounts of NGAL (typically 1:0.1). We used relaxation dispersion experiments (^15^N-labeled CPMG) on this type of mixture to extract information about the bound state of hLCN2-R-NTD through the sharp and intense signal of the free form. For this experiment we used hLCN2-R-NTD-QM as its affinity toward NGAL is identical to the wild type, but its ^1^H,^15^N HSQC spectrum displays sharper and better defined cross-peaks. Additionally, by using the hLCN2-R-NTD-QM mutant we make sure that our measurements are free from conformation exchange caused by the disulfide bridges. We measured ^15^N relaxation dispersion for three different concentrations of NGAL (0.05, 0.1, and 0.15 eq of unlabeled NGAL with respect to hLCN2-R-NTD-QM; Fig. 8, *A* and *B*) and could clearly observe that the exchange contribution to *R*_2_ increased with the concentration of NGAL. For the admixture containing 0.1 and 0.15 eq of NGAL, we analyzed the experimental curves using a two-state exchange formalism (the exchange contribution observed in presence of 0.01 eq of NGAL is too small to be accurately analyzed). We focused on the residues of the C-terminal part (most affected by binding), for which we extrapolated the population of the free and bound form of hLCN2-R-NTD-QM and the exchange rate (*k*_ex_) as global parameters. Additionally, for each residue we could calculate the ^15^N chemical shift difference between the free and bound state (Δω). If our initial assumption is correct, we would expect that the *k*_ex_ and Δω values are independent of the amount of unlabeled NGAL present in the mixture, whereas the population of bound form should increase with increasing NGAL concentration in accordance with the measured affinity.

The *k*_ex_ values were found to be 1300 and 1200 s^−1^ for the mixtures containing 0.1 and 0.15 eq of NGAL, respectively. Additionally, the residue specific Δω values are almost identical for both mixtures (Fig. 8*C*) and are largest around residue 99 (up to 4.5 ppm), indicating important changes in the chemical environment of these residues upon binding. The values for *k_ex_* and Δω tend to indicate that indeed relaxation dispersion gives us access to information about the bound state. However, as can be seen in Fig. 8*D*, the population of bound state is underestimated for both mixtures (as compared with the expected values, knowing the affinity between NGAL and hLCN2-R-NTD). For example, according to the measured affinity, we would expect that in the presence of 0.1 eq of NGAL, nearly 10% of hLCN2-R should be found in its bound state; however, the calculated population is only 1%. This discrepancy could be due to the fact that a two-state exchange formalism does not appropriately describe the properties of the system. Using a three-state exchange formalism did not resolve this discrepancy (data not shown); the calculated *k*_ex_ and Δω remain unchanged as well as the population of free state. The population of the two bound states summed up to the same values as for a single bound state in the two-state exchange formalism. Taken together, this suggests that the system is probably undergoing a complex sequence of binding events that cannot be properly described by this type of simplified formalism.

## Discussion

Because the discovery that NGAL has the special ability to bind siderophores, the siderocalin has attracted a lot of attention. In particular, its apparent pleiotropic functions have been the focus of intensive investigations. Yet, it remains unclear how NGAL can carry out these pleiotropic functions, although its ability to transport iron in and out of the cell seems to be the underlying mechanism supporting NGAL involvement in inflammation, apoptosis, and cancer progression. In that context, it is crucial to understand the interaction between NGAL and its cellular receptor at a molecular level. To gain novel biochemical insights into this interaction, we undertook a biophysical characterization of the interaction between NGAL and the N-terminal domain of its cellular receptor (hLCN2-R-NTD). We first confirmed that hLCN2-R-NTD is located on the extracellular side of the membrane. Next, we were able to express and purify hLCN2-R-NTD and to show, using MS-monitored reduction kinetics, that the sequential formation of two intramolecular disulfide bridges is required to obtain a soluble form of hLCN2-R-NTD. Our ITC measurements, confirmed by our MST and NMR results, show that hLCN2-R-NTD binds apoNGAL with a low micromolar affinity (∼ 10 μm) but do not or poorly bind holo-NGAL. The ability of hLCN2-R-NTD to discriminate between apo- and holo-NGAL corroborates the binding site identified by chemical shift changes mapping (the NGAL calyx).

Additionally, using solution-state NMR, we could show that hLCN2-R-NTD is an IDP, although it retains high degrees of structural heterogeneity, which is in part due to the presence of the two intramolecular disulfide bridges. ^1^H,^15^N HSQC-monitored titrations of hLCN2-R-NTD by unlabeled NGAL revealed that hLCN2-R-NTD binds to NGAL via its relatively hydrophobic C-terminal part and, more surprising, that the formation of the complex leads to severe line broadening for both partners, probably due to the presence of conformational disorder in the complex. Several similar observations have been made for molecular complexes involving one or more disordered partner(s) (45, 46). This type of complex has been named a disordered or “fuzzy” complex (47). The disorder leading to resonance broadening can have various origins. In the case of the intrinsically disordered protein Sic1 and the Cdc4 subunit of an SCF ubiquitin ligase, Sic1 is found in different bound conformations in constant interconversion (45). Whereas in the case of the disordered domain of the Sendai virus nucleoprotein and the C-terminal domain of the phosphoprotein, the disorder is due to a dynamic encounter complex that forms on the surface of the phosphoprotein (46).

We used relaxation dispersion to gain information on the bound state(s), as suggested by the work of Schneider *et al.* (46). Regardless of the formalisms used to treat the experimental data, attempts to fit the data to a theoretical model systematically led to underestimated populations of the bound state(s) compared with the populations expected based on the affinity value.

This suggests the existence of an ensemble of encounter complexes in which the chemical shift differences in the various populated states are minor in contrast to the Sendai virus nucleoprotein and its well defined encounter complex for which the relaxation data could be fitted using a three-state exchange formalism. Additionally, our relaxation dispersion data, regardless of the formalism, suggest that a dominant species exists in the bound state, which could be addressed using this kind of method, and that the C-terminal portion of hLCN2-R-NTD is experiencing important changes in its chemical environment upon binding (large Δω).

Our results are in direct contradiction with the conclusions of Correnti *et al.* (21) who could not observe any direct interaction between NGAL and mLCN2-R. However, it is not clear how these authors controlled 1) the state of their recombinant NGAL (apo- or holo-) and 2) the quality, that is, the formation of the native pattern of disulfide bridges, of their LCN2-R-NTD preparation as the formation of aberrant (non-native) disulfides would probably lead to forms of LCN2-R-NTD that are unable to bind to NGAL. Either or both of these points could explain their inability to observe an interaction between NGAL and mLCN2-R-NTD. Of course, the affinity we measured between hLCN2-R-NTD and NGAL suggests that the N terminus on its own cannot account for the internalization of NGAL by LCN2-R and that other parts of the receptor probably contribute to the interaction. In that context the disordered nature of the complex could be a mechanism that allows a fine-tuning of the interaction between NGAL and its cellular receptor or a biochemical mechanism allowing the receptor to discriminate between apo- and holo-NGAL.

We presented here the first *in vitro* evidences of a physical interaction between NGAL and its putative cellular receptor LCN2-R. Interestingly, we demonstrated that the N-terminal domain of LCN2-R exhibits some specificity toward apoNGAL and forms a peculiar complex that retains significant disorder. Our results represent a first step toward the molecular understanding of the pleiotropic functions of NGAL/LCN2-R.

## Author Contributions

N. C. designed the study and wrote the paper with the help of R. K. and F. T. A.-I. C. M. and K. W. expressed and purified all the protein samples, performed the ITC experiments, and acquired and analyzed the NMR data (except for the sequential assignment). W.-K. L., N. A. W., and F. T. performed the MST experiments and the immunostainings. B. S. and K. B. performed the reduction kinetic. S. Z. and W. K. realized the sequential assignment of hLCN2-R-NTD. All authors analyzed the results and approved the final version of the manuscript.

## Footnotes

3 The abbreviations used are:

NGAL: neutrophil gelatinase-associated lipocalin
HSQC: heteronuclear single quantum coherence
ITC: isothermal titration calorimetry
LCN2: lipocalin 2
LCN2-R: LCN2 cellular receptor
LCN2-R-NTD: N-terminal domain of the lipocalin 2 receptor
mLCN2: mouse LCN2
rLCN2: rat LCN2
MST: microscale thermophoresis
Ent: enterobactin
TM: transmembrane
TEV: tobacco etch virus
ESI: electrospray ionization
IDP: intrinsically disordered protein
CPMG: Carr-Purcell-Meiboom-Gill.
